# A Supramolecular Nanoparticle of Pemetrexed Improves the Anti-Tumor Effect by Inhibiting Mitochondrial Energy Metabolism

**DOI:** 10.3389/fbioe.2021.804747

**Published:** 2021-12-21

**Authors:** Hui Liu, Chunlei Guo, Yuhong Shang, Lin Zeng, Haixue Jia, Zhongyan Wang

**Affiliations:** ^1^ Henan Key Laboratory of Immunology and Targeted Drug, Henan Collaborative Innovation Center of Molecular Diagnosis and Laboratory Medicine, School of Laboratory Medicine, Xinxiang Medical University, Xinxiang, China; ^2^ ChosenMed Technology Co., Ltd., Beijing, China; ^3^ Tianjin Key Laboratory of Radiation Medicine and Molecular Nuclear Medicine, Institute of Radiation Medicine, Chinese Academy of Medical Sciences and Peking Union Medical College, Tianjin, China

**Keywords:** supramolecular nanoparticle, pemetrexed, metabolism, mitochondrial, tumor therapy

## Abstract

In recent years, supramolecular nanoparticles consisting of peptides and drugs have been regarded as useful drug delivery systems for tumor therapy. Pemetrexed (PEM) is a multitarget drug that is effective for many cancers, such as non-small cell lung cancer. Here, RGD-conjugated molecular nanoparticles mainly composed of an anticancer drug of PEM (PEM-FFRGD) were prepared to deliver PEM to tumors. The peptide could self-assemble into a nanoparticle structure with diameter of about 20 nm. Moreover, the nanoparticle showed favorable solubility and biocompatibility compared with those of PEM, and the MTT test on A549 and LLC cells showed that the PEM-FFRGD nanoparticles had stronger cytotoxic activity than PEM alone. Most importantly, the nanoparticle could promote tumor apoptosis and decrease mitochondrial energy metabolism in tumors. *In vivo* studies indicated that PEM-FFRGD nanoparticles had enhanced antitumor efficacy in LLC tumor-bearing mice compared to that of PEM. Our observations suggested that PEM-FFRGD nanoparticles have great practical potential for application in lung cancer therapy.

## Introduction

Lung cancer is the main cause of death worldwide, with approximately 1 million cases being recorded each year (Ferlay et al., 2019). Pemetrexed (PEM) is a multitarget antifolate drug used for cancer treatment (Adjei, 2003) and it was approved by the FDA to treat malignant pleural non-small cell lung cancer (NSCLC) as well as mesothelioma in 2004 (Manegold et al., 2009). Additionally, it was reported that PEM had a therapeutic effect on colorectal tumors and lung cancer (Hanauske et al., 2001; Schaer et al., 2019). It was reported that PEM could hinder translation inhibition upon low glucose in NSCLC, and PEM combined with 2-Deoxy-glucose showed enhanced efficacy in decreasing cell proliferation of Malignant Pleural Mesothelioma (Piecyk et al., 2021). These finds suggest that PEM may have relationship with glucose metabolism. However, there is few reports studies the role of PEM on glucose metabolism. what’s more, there are some limits to the clinical use of PEM, such as fast clearance, low water solubility, low bioavailability, poor targeting selectivity and penetration toward the tumor, and potential spleen and kidney toxicity (Li et al., 2007; Glezerman et al., 2011). Hence, it is necessary to further investigate and improve the antitumor efficiency of PEM.

In recent years, nano-delivery systems, such as poly (lactic-co-glycolic acid)/polylactic acid nanoparticles, gold/silica nanomaterials, polymeric nanoparticles, and hyaluronan, have proven to be promising in drug delivery to improve the therapeutic effect (Liang et al., 2009; Liang et al., 2014; Cai et al., 2017; Amano et al., 2019; Sun et al., 2019; Naskar et al., 2021). Moreover, self-assembling peptide nanoparticles displayed huge prospects in the delivery of antitumor drug due to their high loading capacity, good biocompatibility, inherent degradability, controllable drug release and easy preparation (Cheetham et al., 2013; Basu et al., 2016; Zhang et al., 2016). They can be used as carriers both for physical encapsulation of drugs (Wu et al., 2015; Sato et al., 2018) and for chemical conjugation among drug molecules and self-assembling peptides (Ren et al., 2014; Yang et al., 2019). The synthesized peptide-drug compounds can effectively improve the solubility and stability of hydrophobic drugs under physiological conditions and enhance the accumulation and retention of drugs in tumor tissues (Gao et al., 2018; Qi et al., 2018; Yang et al., 2019). Currently, a variety of antitumor peptide-drug conjugates that form injectable nanoparticles have been constructed and have shown intensive antitumor efficacy *in vitro* and *in vivo* (Murphy et al., 2008; Gao et al., 2009; Song et al., 2013). For example, PEM-FE conjugates have been reported to have better cytotoxic efficacy than the free PEM (Lock et al., 2017). However, the therapeutic evaluations and mechanism is not further studied, and whether the PEM-FE can specific deliver PEM to tumors is not found. Therefore, further optimization and investigation on peptide-based nanoparticles to deliver PEM is promising.

Many surface receptors that are overexpressed in cancer cells have been used for targeted delivery by nanoparticle. αvβ3 integrin is one receptor that is absent in normal tissues but highly expressed in many tumors, thus making it a suitable target for selective delivery (Chen and Chen, 2011). αvβ3 integrins can interact with the Arg-Gly-Asp (RGD) motif existed in a majority of extracellular matrix proteins (Xiong et al., 2002; Graf et al., 2012). Several studies have suggested that the nanoparticle (NP) system modified by RGD can effectively target tumors (Wang et al., 2014; Zhao et al., 2014; Hou et al., 2016; Yadav et al., 2020). However, only a few studies have reported the targeted delivery of PEM (Lock et al., 2017). Therefore, RGD-conjugated nanoparticle delivery systems show great promise in cancer therapeutics.

Besides, aromatic motif phenylalanine-phenylalanine (FF) also often is used for constructing suparmolecular nanomaterials to improve self-assembly capability and regularize molecular arrangement (Ryan et al., 2015; Diaferia et al., 2019; Gallo et al., 2021; Li et al., 2021). In this study, we fabricated an RGD peptide-conjugated, self-assembling, and FF-based supramolecular nanoparticle mainly formed by PEM to concurrently enhance active targeting and anticancer efficiency toward tumors. As the self-assembly peptides are promising organic materials, which are widely applied in tissue engineering, drug delivery systems and biomaterials for they showed improved mechanical properties, stability, hydrophilicity and good biocompatibility (Ryan et al., 2015; Diaferia et al., 2019; Gallo et al., 2021; Li et al., 2021). PEM-FFRGD were synthesized with a method of standard solid phase peptide synthesis, and the PEM-FFRGD monomers can easily form into particle-like nanostructure via PH adjust and ultrasound method. Moreover, the prepared nanoparticles can not only prominently enhance the solubility of PEM but also promote tumor apoptosis and decrease energy metabolism. As a result, this PEM-FFRGD nanoparticle boosted antitumor activity *in vitro* and *in vivo* compared with that of PEM alone. This supramolecular nanoparticle may provide a unique PEM delivery system for targeted therapy of lung cancer.

## Materials and Methods

### Materials

2-Cl-trityl chloride resin was supplied by Nankai University Resin Co., Ltd. Fmoc-amino acids and O-benzotriazole-N,N,N′,N′-tetramethyluronium-hexafluorophosphate (HBTU) were purchased from GL Biochem (Shanghai, China). PEM and the other starting materials were supplied by Aladdin. Dulbecco’s modified Eagle’s medium (DMEM), fetal bovine serum (FBS) and penicillin/streptomycin were obtained from HyClone. DAPI (4′, 6-diamidino-2-phenylindole) and 3-(4,5-dimethyl-2-thiazolyl)-2,5-diphenyl-2H-tetrazolium bromide (MTT) were purchased from Solarbio (Beijing, China). A mitochondrial membrane potential detection kit and reactive oxygen detection kit were supplied by Beyotime Biotechnology, and a Seahorse XF Glycolysis Stress Test Kit was supplied by Agilent.

### Animals

C57BL/6 mice (6–8 weeks) were supplied by Vital River Laboratory Animal Technology Co., Ltd. (Beijing, China). All mice were maintained under specific pathogen-free conditions in the animal facility of Xinxiang Medical University. The Xinxiang Medical University Experimental Animal Ethics Committee approved all animal experiments.

### Synthesis and Characterization of PEM-FFRGD Conjugate

The designed peptide PEM-FFRGD was synthesized using a method of standard solid-phase peptide synthesis (SPPS) by using 2-chlorotrityl chloride resin and N-Fmoc protected amino acids. The crude PEM-FFRGD conjugates were purified through reversed-phase high-performance liquid chromatography (HPLC). LC-MS (Shimadzu, 2020) was utilized to characterize the PEM-FFRGD conjugates.

### Preparation and Characterization of PEM-FFRGD Nanoparticle

The peptide (0.5 wt%) was dispersed in phosphate buffer solution (PBS), and sodium carbonate was added to adjust the pH of the solution to 7.4 followed by an ultrasound for self-assembly. The laser was used to identify aggregation of PEM-FFRGD. The nanostructures of self-assembled PEM-FFRGD were viewed using transmission electron microscopy (TEM, HITACHI HT7700 Exalens) following a negative staining technique.

### Cytotoxicity Analysis

Lewis lung cancer (LLC) and A549 cells (100 µl) were plated at a concentration of 5 × 103 cells/well in a flat-bottomed 96-well plate on Day 0. On day 1, cells were exposed to different concentrations of PEM and PEM-FFRGD for 72 h. Then, the culture medium was removed, and MTT (0.5 mg/ml) was added to each well and incubated for 4 h. The supernatant was discarded, and dimethyl sulfoxide (100 µl) was used to completely dissolve the crystals. Absorbance at 490 nm was detected using a microplate reader (EXL-800; Bio-Tek, Winooski, VT, United States).

### Live/Dead Assay

LLC cells and A549 cells (5,000 cells/well) were plated in 96-well plates and cultured for 24 h. Then, the cells were cocultured with 6 and 12 µM PEM and PEM-FFRGD for 72 h, respectively. The cells were washed with PBS and stained with calcein-AM and ethidium homodimer-1 (EthD-1) for 15 min. Then, the cells were viewed under microscopy (TI-S, Nikon, United States).

### Flow Cytometry Analysis of Apoptosis

LLC cells and A549 cells (1.0 × 105 cells/well) were cultured in 12-well plates for 24 h, and then LLC cells and A549 cells were treated with 6 and 12 µM PEM and PEM-FFRGD for another 72 h, respectively. The cells were then collected and stained with annexin V and PI for 20 min at room temperature and analyzed using flow cytometry.

### Detection of ROS Levels

To detect reactive oxygen species (ROS), 2 × 105 LLC and A54 cells were plated in 24-well plates and cultured for 24 h. Then, the cells were treated with 6 and 12 µM PEM and PEM-FFRGD for 48 h, respectively, following which the cells were stained with DCFH-DA (10 µM) for 20 min with CCCP treatment (10 μM) as a positive control. The cells were washed and resuspended in PBS samples after staining and then analyzed using flow cytometry.

### Measurement of Mitochondrial Membrane Potential

The mitochondrial membrane potential (∆Ψm) was measured using a mitochondrial detection kit (Beyotime, Shanghai, China). LLC and A549 cells were cultured in 24-well plates on coverslips at 5 × 104 cells/well and incubated for 12 h. Then, PEM and PEM-FFRGD (6 µM) were added and incubated with LLC cells, and the A549 cells were incubated with 12 µM PEM-FFRGD and PEM. After 48 h, the nuclei of the cells were stained with JC-1 and DAPI, followed by visualization using confocal laser scanning microscope (CLSM).

### Metabolic Assessments

ECAR detection was performed using an XF24e Extracellular Flux analyzer (Agilent). Cells with different treatments were seeded on an XF24 microplate at 10,0000 cells/well 1 day prior to the assay and cultured in a cell incubator with CO_2_ for 24 h. When glucose, oligomycin, and 2-DG were loaded into the corresponding injection port of sensor cartridges and the utility plate was placed on the instrument tray for calibration, the cell microplates were placed into a 37°C incubator without CO_2_ for 45–60 min prior to running with warmed detection medium at 500 μl/well. Then, the cell culture microplate was loaded on the instrument following calibration. Wave 2.4 software (Agilent) was applied to analyze the data.

### *In vivo* Antitumor Efficacy

C57BL/6J mice were subcutaneously inoculated with Lewis lung tumors and then randomly divided into four groups (5 mice per group) when the tumor sizes were approximately 100 mm^3^. Mice in Groups 1 and 2 received PBS and FFRGD gels through i.v. injections, while mice in Groups 3 and 4 were administered 20 mg/kg PEM and PEM-FFRGD nanoparticles by i.v. injection. Repeated injections were administered on Days 1 and 5. Tumor size and body weight were monitored every 2 days, and the tumor volume was calculated according to the following formula: tumor volume (V, mm^3^) = (tumor width)^2^ × (tumor length)/2.

### Statistical Analysis

GraphPad Prism 8.0 was applied for statistical analyses. All data are presented as the means ± SD. Statistical analysis was acquired using the Student’s t-test, and a difference between groups of *p* < 0.05 was considered significant.

## Results and Discussion

### Preparation and Characterization of PEM-FFRGD Nanoparticle

**FIGURE 1:**
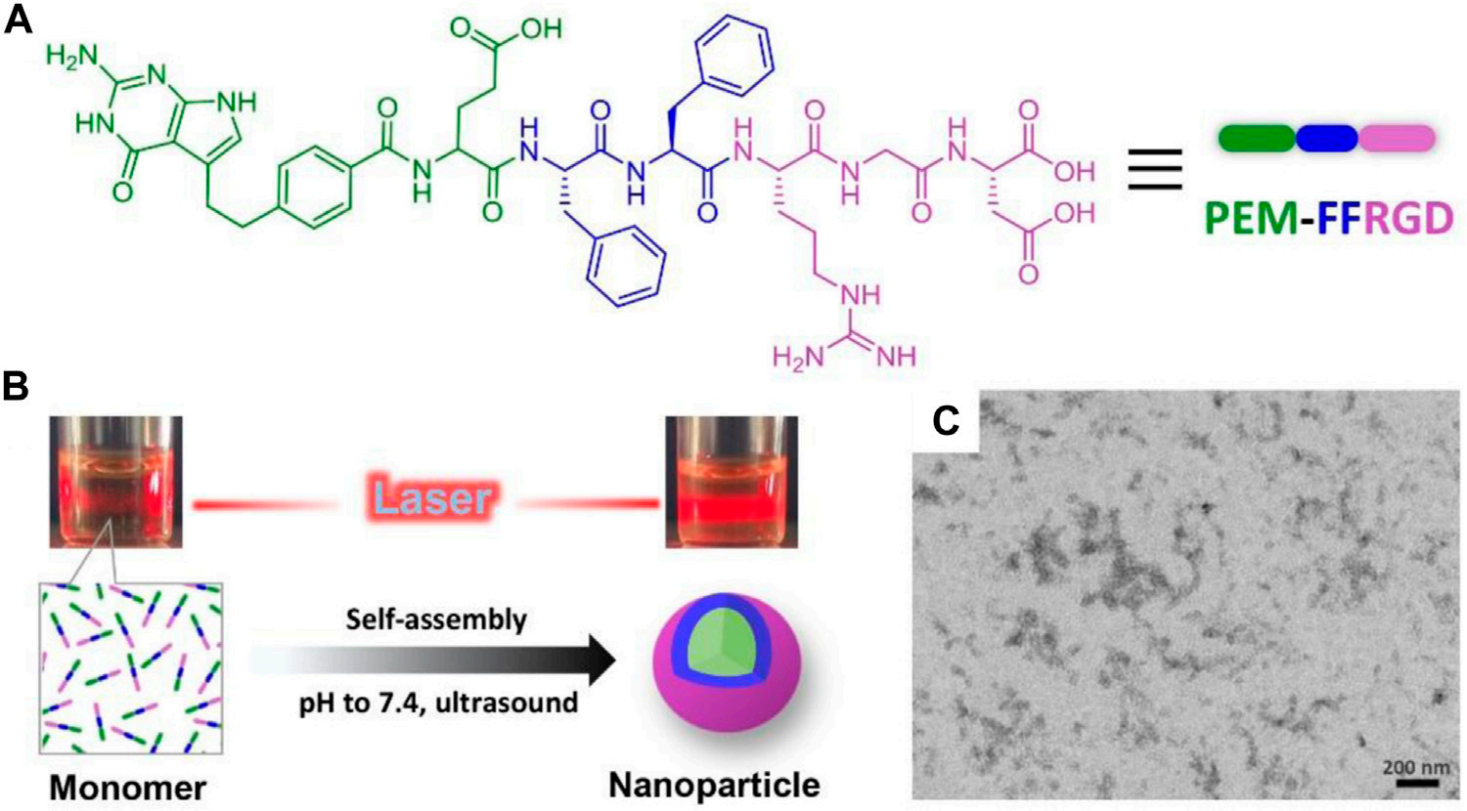
Synthesis and characterization of PEM-FFRGD nanoparticle. **(A)** Chemical structure of the PEM-FFRGD molecule, **(B)** The solutions under laser light of PEM-FFRGD monomer at pH = 5 and PEM-FFRGD nanoparticle at pH = 7.4, respectively, **(C)** TEM images of PEM-FFRGD nanoparticle.

We recently developed a self-assembling peptide (PEM-FFRGD) mainly contained PEM, which was capable of forming supramolecular nanoparticles (Figure 1A) and avoiding the non-specific interactions with normal tissues. The concept of self-assembling peptide-drug compounds is one in which the drug loading capacity can be precisely held at the level of molecular design (Su et al., 2015; Ma et al., 2016). We intended to further investigate its application in treatment of tumors. In this component, the PEM drug loading in PEM-FFRGD conjugates was 39 wt%, which was calculated as the ratio of the molecular mass of PEM to PEM-FFRGD conjugate (Supplementary Figure S1). Moreover, the conjugate structures were confirmed by LC-MS (Supplementary Figure S2). The m/z value of PEM-FFRGD was found to be 1,050.4 in accordance with the prospective exact masses of the compounds.

We dispersed the PEM-FFRGD compound in phosphate-buffered saline (PBS; pH = 7.4) at a concentration of 0.5 wt%, and a transparent solution was formed after adjusting pH to 7.4 and an ultrasound operation (Figure 1B). The solution exhibited an obvious Tyndall effect compared with the solution of PEM-FFRGD monomer, indicative of the formation of nanoaggregate (Figure 1B). Then, the microstructure of the self-assembled PEM-FFRGD was detected using TEM. The results showed that the PEM-FFRGD compound formed nanoparticles with diameters of approximately 20 nm (Figure 1C).

### PEM-FFRGD Enhanced the Cytotoxicity Activity

**FIGURE 2:**
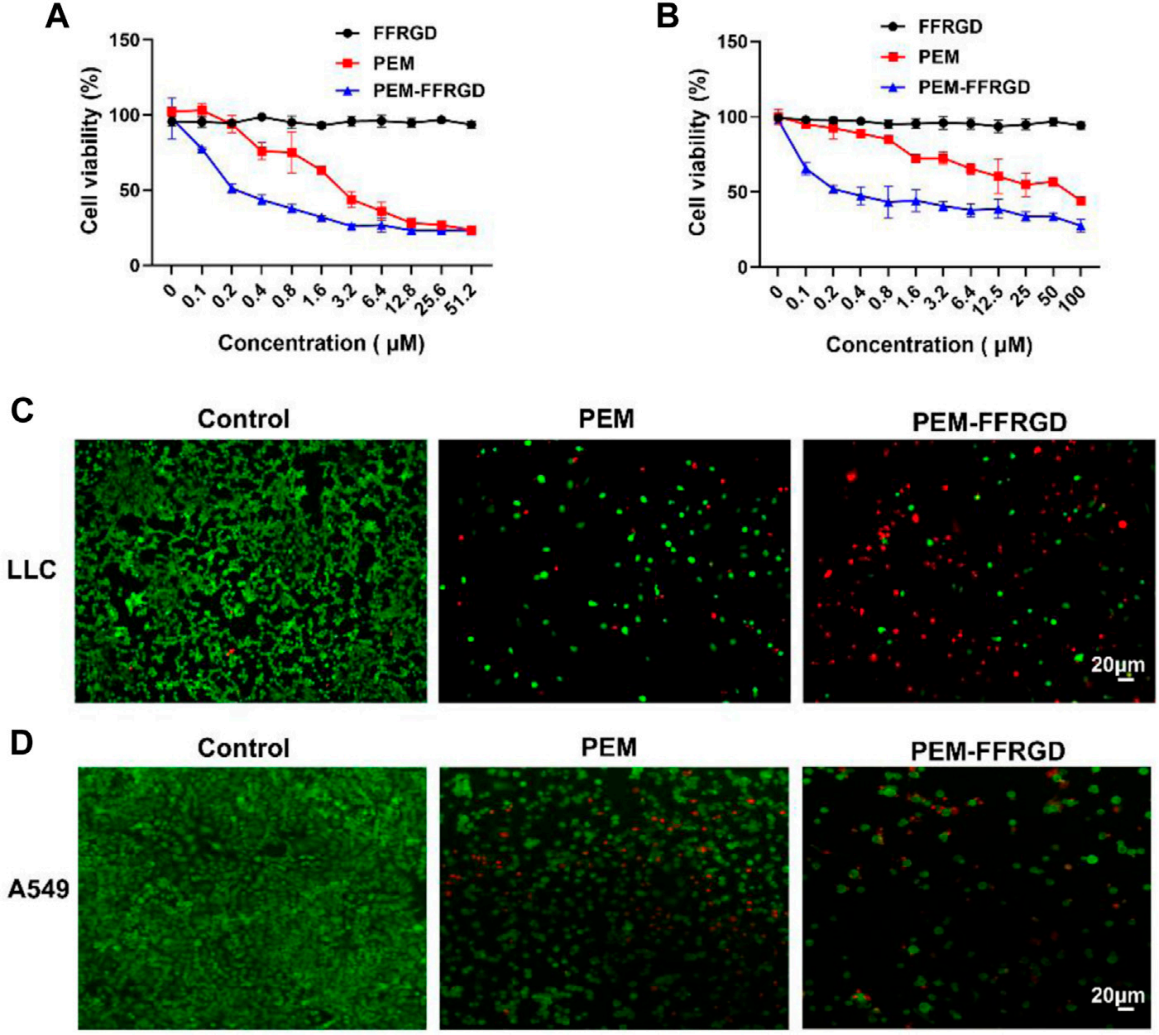
The cytotoxicity of the PEM-FFRGD nanoparticle was determined using MTT and live/dead assays. **(A)** LLC cells, **(B)** A549 cells were cultured with a series of concentrations of FFRGD, PEM and PEM-FFRGD for 72 h, and then the cell viability was examined using MTT assays, **(C,D)** LLC cells and A549 cells were exposed to 6 and 12 μM FFRGD, PEM and PEM-FFRGD for 72 h and then imaged using microscopy. Scale bars, 20 µm.

To investigate the cytotoxic activity of the PEM-FFRGD nanoparticle *in vitro*, various standard MTT viability assays were performed on A549 and LLC cells, with PEM and FFRGD as controls. As shown in Figure 2A, PEM-FFRGD showed stronger cytotoxic activity toward A549 and LLC cells than PEM alone. The viability of LLC cells was 50% after treatment with 0.2 µM PEM-FFRGD, but the viability was decreased to 26% following treatment with 6 µM PEM-FFRGD. Similar results were acquired for A549 cells exposed to PEM-FFRGD (Figure 2B). The PEM-FFRGD and PEM showed no cytotoxicity when LLC and A549 cells were incubated with different concentrations of PEM-FFRGD and PEM for 24 h. When the incubation time was extended to 48 h, the PEM-FFRGD showed cytotoxic activity toward LLC and A549 cells and the viability of cells treated with PEM-FFRGD is lower than treated with PEM (Supplementary Figure S3). Similar results were acquired by live/dead assays (Supplementary Figure S4). These results may be attributed to the RGD motif on PEM-FFRGD as a result of more accumulation by tumor cells. Our results also suggested that 100 μM FFRGD did not display any distinct cell toxicity, indicating that the PEM-FFRGD nanoparticle has good biocompatibility.

We further detected the cytotoxic activity of PEM and PEM-FFRGD by live/dead staining. A549 and LLC cells were plated into 96-well plates and exposed to 12 and 6 μM PEM-FFRGD, and PEM was used as a control. After coculture for 72 h, the cells were stained with calcein-AM (green) and EthD-1 (red) and visualized using a microscope. As displayed in Figures 2C,D, PEM-FFRGD killed most A549 and LLC cells, with a few green signals observed. Additionally, PEM could kill most cells but exhibited more green fluorescence specific to living cells than PEM-FFRGD. These results indicate that PEM-FFRGD has stronger cytotoxicity against A549 and LLC cells, which may be due to the sustained release of PEM from the PEM-FFRGD Gel.

### Apoptotic Mechanism of PEM-FFRGD

**FIGURE 3:**
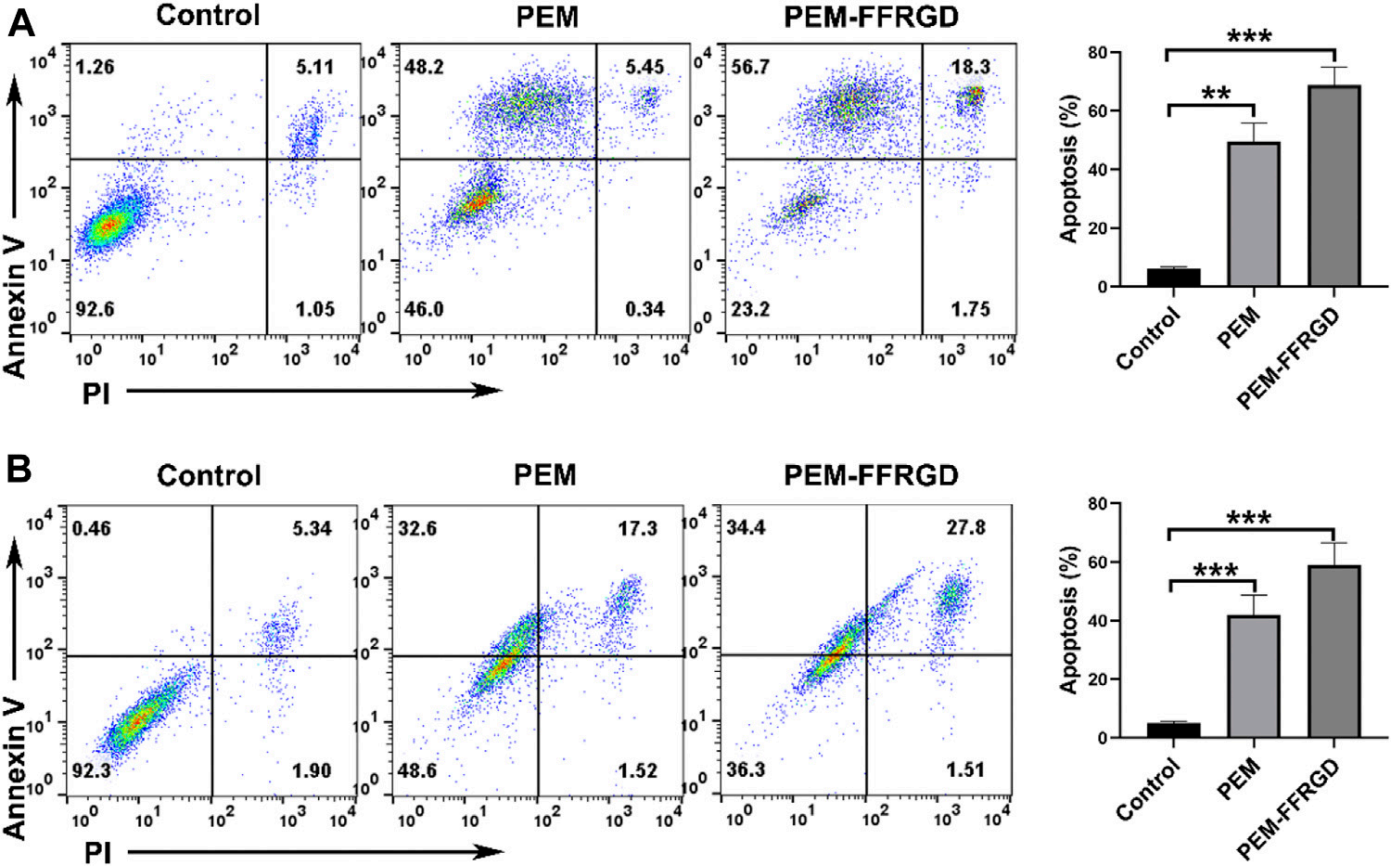
Apoptotic mechanism of PEM-FFRGD detected using flow cytometry. LLC cells **(A)** and A549 cells **(B)** were incubated with 6 and 12 μM FFRGD, PEM and PEM-FFRGD for 72 h, stained with Annexin V/PI and detected using flow cytometry.

Annexin V/PI staining was used to investigate the influences of PEM-FFRGD on the apoptosis of LLC and A549 cells. As shown in Figure 3, both PEM-FFRGD nanoparticles and PEM induced significant apoptosis signals in LLC and A549 cells when the cells were incubated with these drugs. In addition, PEM-FFRGD induced stronger apoptosis in either LLC or A549 cells than PEM, with killing rates of 73 and 62%, respectively.

### PEM-FFRGD Suppresses Energy Metabolism

**FIGURE 4:**
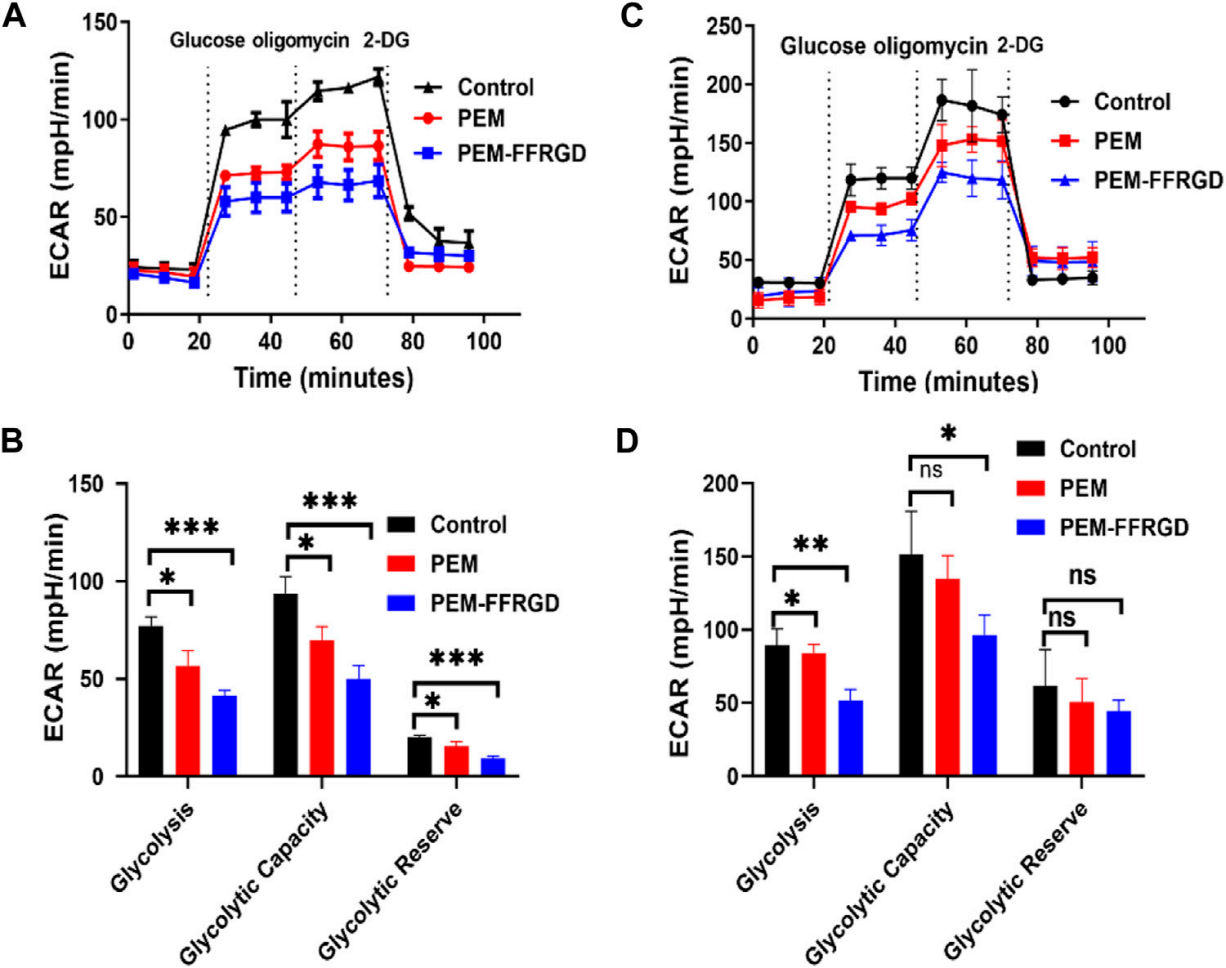
PEM-FFRGD suppresses energy metabolism. LLC cells **(A,B)** and A549 cells **(C,D)** were incubated with 6 and 12 μM FFRGD, PEM and PEM-FFRGD for 24 h, and then the extracellular acidification rate was detected using an XF24e extracellular flux analyzer.

Tumor cells can reprogram their metabolism to meet their bioenergy and biosynthetic needs, and increased glycolysis is a main biochemical feature of tumors (Ma et al., 2021). Tumor glycolysis plays vital role in the tumor rapid growth and cancer metastasis because it provides energy (Elia et al., 2018). Therefore, we detected the effect of PEM-FFRGD on glycolytic metabolism in LLC and A549 cells using an XF24e extracellular flux analyzer. We found that the extracellular acidification rate and glycolysis level of PEM-FFRGD-treated LLC cells were remarkably lower than those of the PEM group and control group (Figures 4A,B). Moreover, the glycolytic capacity and glycolytic reserve were also lower than those of the control group and PEM group. Similar results were found for A549 cells exposed to PEM-FFRGD (Figures 4C,D). These data suggest that PEM-FFRGD nanoparticles are more favorable for regulating the metabolic demands of tumor cells and thus suppressing tumor cell growth and proliferation.

### PEM-FFRGD Decreases the Level of Intracellular ROS Production and Mitochondrial Membrane Potential

**FIGURE 5:**
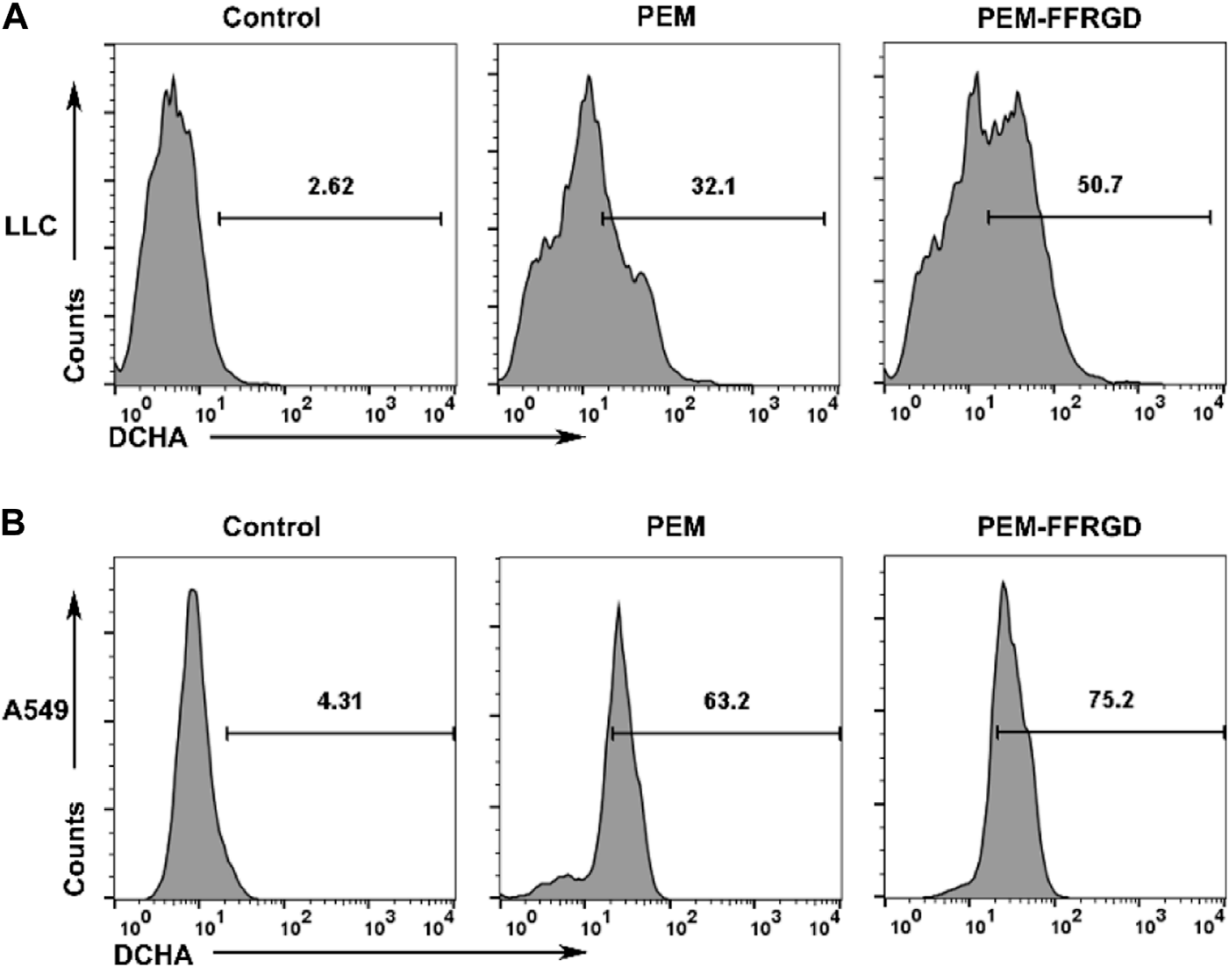
Function of mitochondria assessed using flow cytometry. LLC cells **(A)** and A549 cells **(B)** were incubated with 6 and 12 μM FFRGD, PEM and PEM-FFRGD for 48 h, and then the intracellular ROS levels were measured using the ROS assay kit.

Previous results suggested that PEM-FFRGD regulated the energy metabolism of LLC and A549 cells, and the main place of energy metabolism was the mitochondria. Damage to mitochondrial function or the electron transport chain will directly lead to an increase in ROS levels in cells. To further explore the mechanism of the effect of PEM-FFRGD on energy metabolism, we detected the effect of PEM-FFRGD on intracellular ROS levels using the ROS assay kit. As shown in Figures 5A,B, after LLC and A549 cells were cultured with PEM-FFRGD and PEM for 48 h, the amount of ROS produced in PEM-FFRGD-treated cells was distinctly higher than that in PEM-treated cells, indicating that PEM-FFRGD was more beneficial for mediating ROS production. These results are strongly identical to the cytotoxicity activity data acquired from the MTT test and live/dead assay.

**FIGURE 6:**
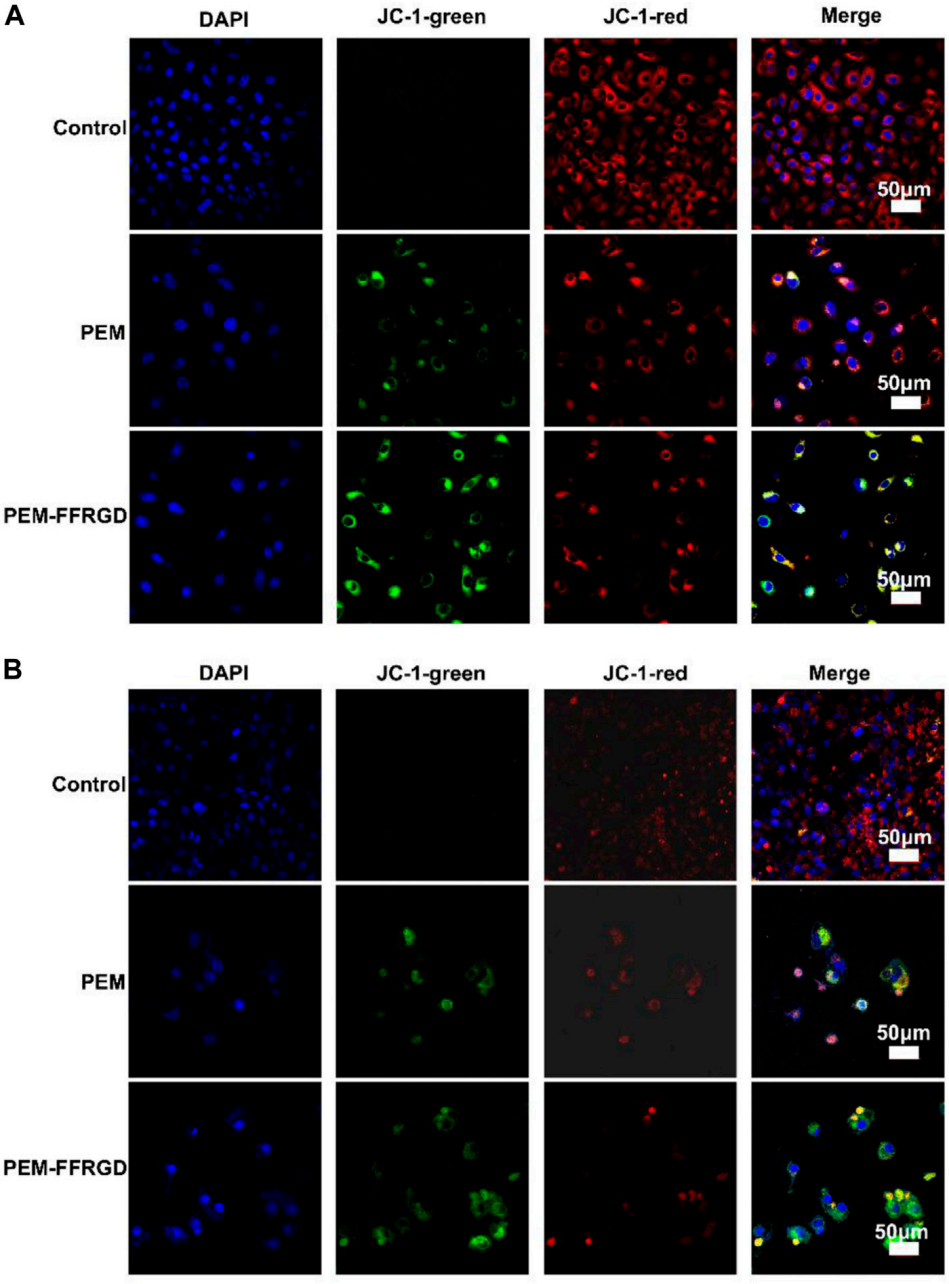
PEM-FFRGD affects mitochondrial membrane potential. LLC cells **(A)** and A549 cells **(B)** were incubated with 6 and 12 μM FFRGD, PEM and PEM-FFRGD for 48 h, and then the mitochondrial membrane potential was imaged by confocal microscopy.

In addition, a specific fluorescent probe was used to detect mitochondrial membrane potential to verify the influence of PEM-FFRGD on mitochondrial respiration. The results showed that PEM-FFRGD could more significantly decrease mitochondrial membrane potential in both LLC and A549 cells than the PEM group and control group (Figures 6A,B). In summary, PEM-FFRGD induced ROS accumulation and decreased mitochondrial membrane potential in A549 and LLC cells.

### *In vivo* Efficacy of the PEM-FFRGD in an LLC Xenograft Model

**FIGURE 7:**
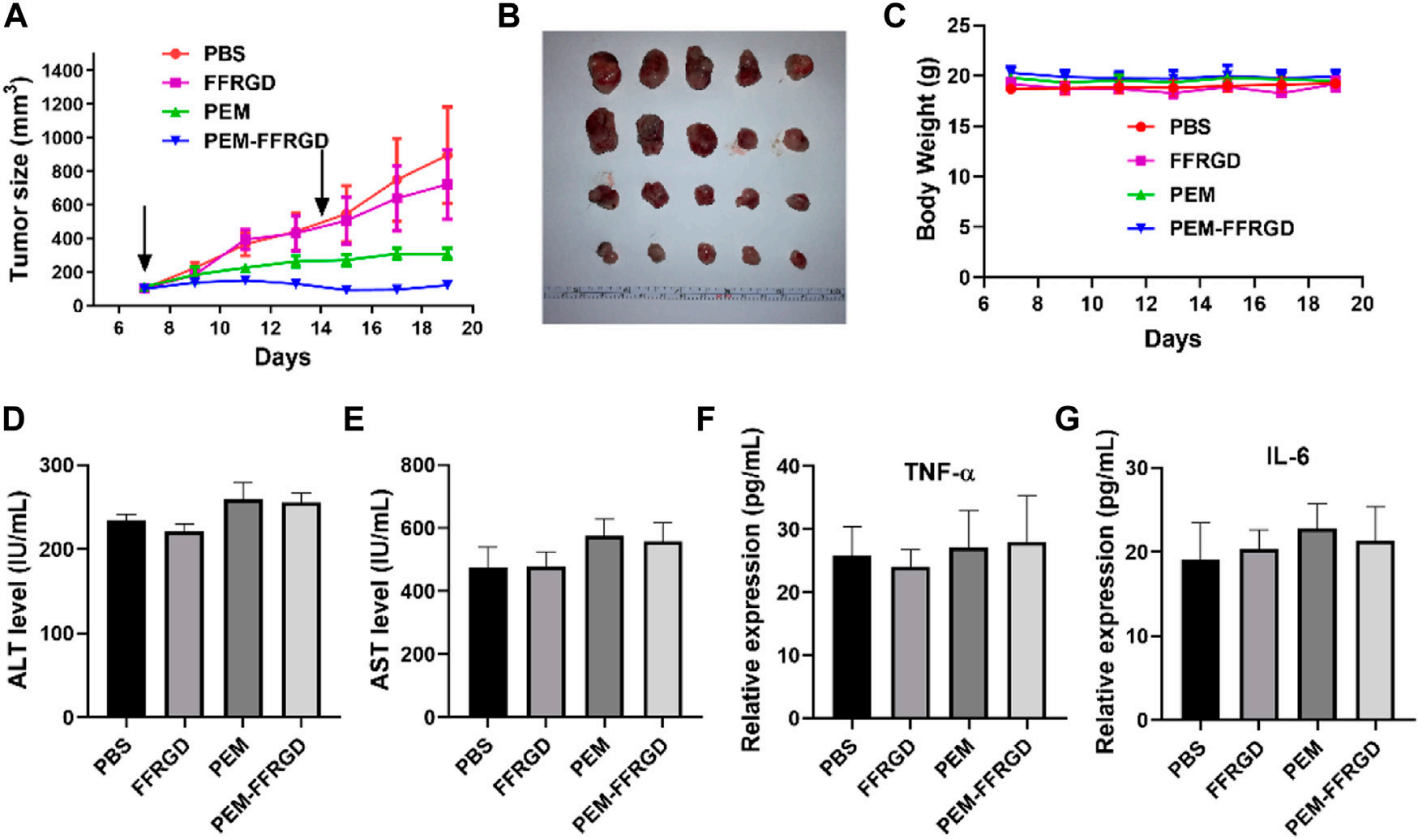
*In vivo* antitumor activity of PEM-FFRGD. **(A)** Tumor growth curves of LLC tumor-bearing mice after intravenous injection of the conjugates at a dose of 20 mg/kg, **(B)** Image of tumors, **(C)** Body weight of different groups, **(D)** ALT level in serum, **(E)** AST level in serum, **(F)** TNF-α level in serum, and **(G)** Il-6 level in serum. Data are expressed as the means ± SEM (*n* = 5).

The efficacy of the fabricated PEM-FFRGD was further investigated as therapy for LLC tumors in C56B6/J mice *in vivo*. LLC tumors were injected under the skin of the mice, and then PEM-FFRGD and PEM were administered intravenously when the tumor volume reached approximately 100 mm^3^ on Day 1 and Day 7. The effect of the different drugs on tumor growth was assessed by measuring the tumor volumes within 2 weeks. The tumors in the control group and Gel alone group grew very rapidly, while tumor growth in the PEM-FFRGD and PEM groups was significantly inhibited. Moreover, compared with the PEM group, the tumor volume of the PEM-FFRGD-treated group was less (Figures 7A,B). No distinct differences in the weight of the mice was observed among the groups (Figure 7C), which suggests that not only Gel but also PEM had no influence on mouse weight. In addition, we examined the serum levels of TNFα, IL-6, ALT and AST in the mice. The results suggested that the levels of TNFα, IL-6, ALT and AST in mice treated with PEM-FFRGD were not different from those in mice treated with PBS and PEM alone (Figures 6G, 7D). These results implied that PEM-FFRGD has no systemic toxicity in mice.

## Conclusion

In summary, we developed a PEM-based theranostic filament nanoparticle system (PEM-FFRGD) that could target the tumor site by RGD motifs. Our results showed that the nanoparticle not only significantly inhibited LLC and A549 growth *in vitro* but also suppressed LLC growth *in vivo*. This was accompanied by favorable biocompatibility, leading to synergistic suppression of cell proliferation, promotion of tumor apoptosis and decrease of mitochondrial energy metabolism of tumors by enhancing the level of intracellular ROS production and abating mitochondrial membrane potential. The conjugate PEM-FFRGD could sustainably release PEM and displayed great advantages and powerful antitumor efficacy in targeting LLC xenograft tumors on the basis of the inhibition of energy metabolism and tumor-targeted administration *in vitro* and *in vivo*. The improvement of antitumor activities is mainly attributed to the RGD and FF peptides, which release the PEM slowly and prevent normal tissue from nonspecific uptake of PEM. These data indicate that the PEM-FFRGD conjugate offered a strategy to effectively target the presentation of PEM and RGD peptides to LLC cells and restrain tumor growth in a synergistic manner.

## Data Availability

The original contributions presented in the study are included in the article/Supplementary Material, further inquiries can be directed to the corresponding author.
