# Supplementary material for: A Supramolecular Nanoparticle of Pemetrexed Improves the Anti-Tumor Effect by Inhibiting Mitochondrial Energy Metabolism
Source: Front Bioeng Biotechnol. 2021 Dec 21;9:804747. doi: 10.3389/fbioe.2021.804747 (PMC8724251; doi:10.3389/fbioe.2021.804747)
Supplement: Supplementary file 1 [file DataSheet1.docx]

A supramolecular nanoparticle of pemetrexed improves the anti-tumor effect by inhibiting mitochondrial energy metabolism

*Hui Liu^1†^, Chunlei Guo^1†^, Yuhong Shang^2†^, Lin Zeng^1^, Haixue Jia^3^, Zhongyan Wang^3*^*

^1^Henan Key Laboratory of Immunology and Targeted Drug, Henan Collaborative Innovation Center of Molecular Diagnosis and Laboratory Medicine, School of Laboratory Medicine, Xinxiang Medical University, Xinxiang 453003, Henan, China

^2^ChosenMed Technology Co. Ltd, Beijing, China

^3^Tianjin Key Laboratory of Radiation Medicine and Molecular Nuclear Medicine, Institute of Radiation Medicine, Chinese Academy of Medical Sciences &Peking Union Medical College, Tianjin 300192, P.R. China.

*** Correspondence:**Corresponding Author
wangzhongyan@irm-cams.ac.cn

*^†^* These authors contributed equally to this work

**Keywords:** supramolecular nanoparticle, pemetrexed, metabolism, tumor therapy.

**S1. Drug loading for PEM-FFRGD**

PEM mass = 427.4 g/mol – 17 g/mol (-OH) = 410.4 g/mol

PEM-FFRGD mass = 1050.4 g/mol

PEM-FFRGD drug loading = 410.4/1050.4 *100% = 39.0%

**S2. LC-MS of** **PEM-FFRGD**


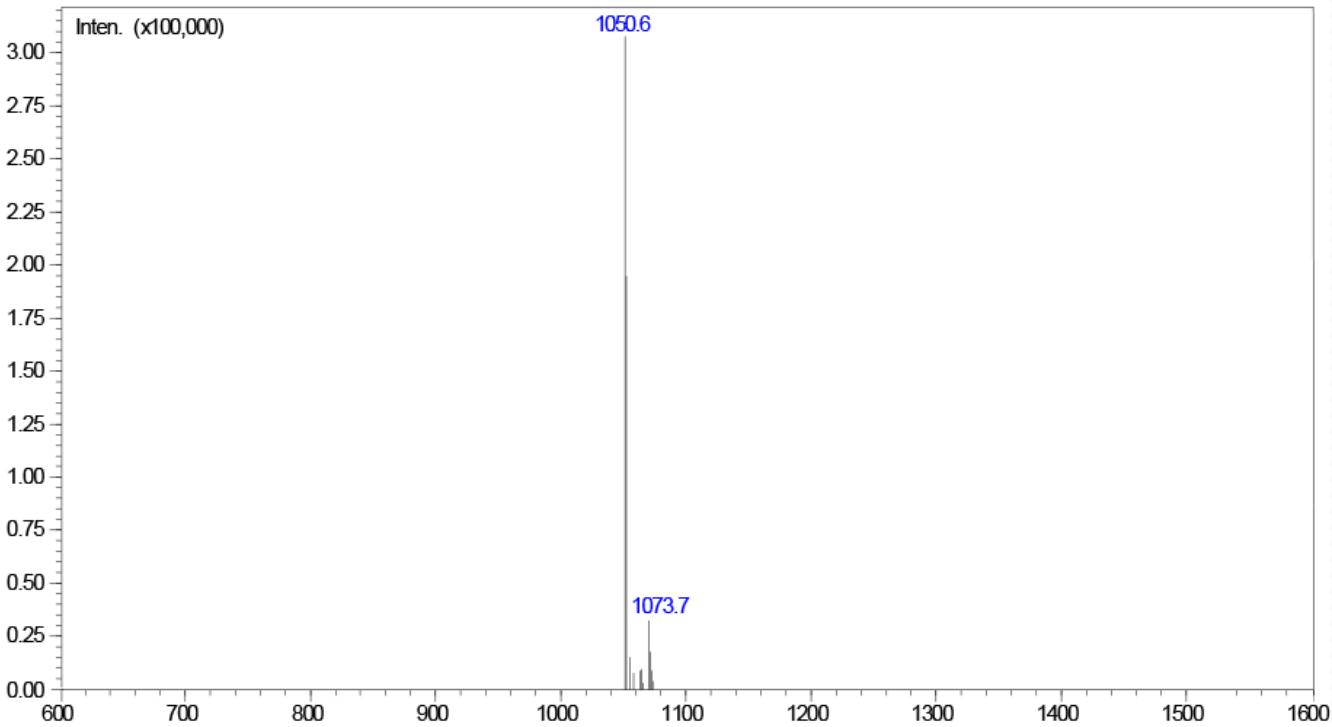


**S3.** **The cytotoxicity of the PEM-FFRGD nanoparticle was determined using MTT**


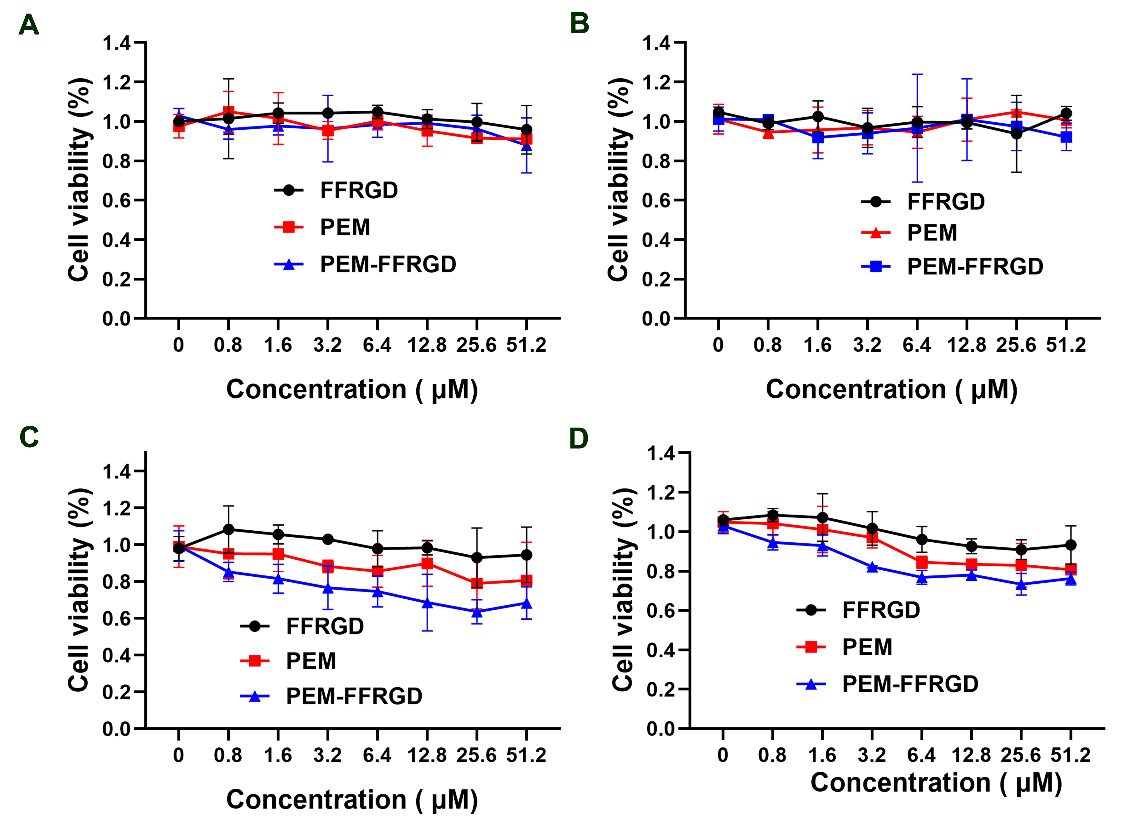


**FIGURE S3∣**The cytotoxicity of the PEM-FFRGD nanoparticle was determined using MTT assays. **(A)** LLC cells, **(B)** A549 cells were cultured with a series of concentrations of FFRGD, PEM and PEM-FFRGD for 24h, and then the cell viability was examined using MTT assays, **(C)** LLC cells, **(D)** A549 cells were cultured with a series of concentrations of FFRGD, PEM and PEM-FFRGD for 48h, and then the cell viability was examined using MTT assays.

**S4. The cytotoxicity of the PEM-FFRGD nanoparticle was determined using live/dead assays**


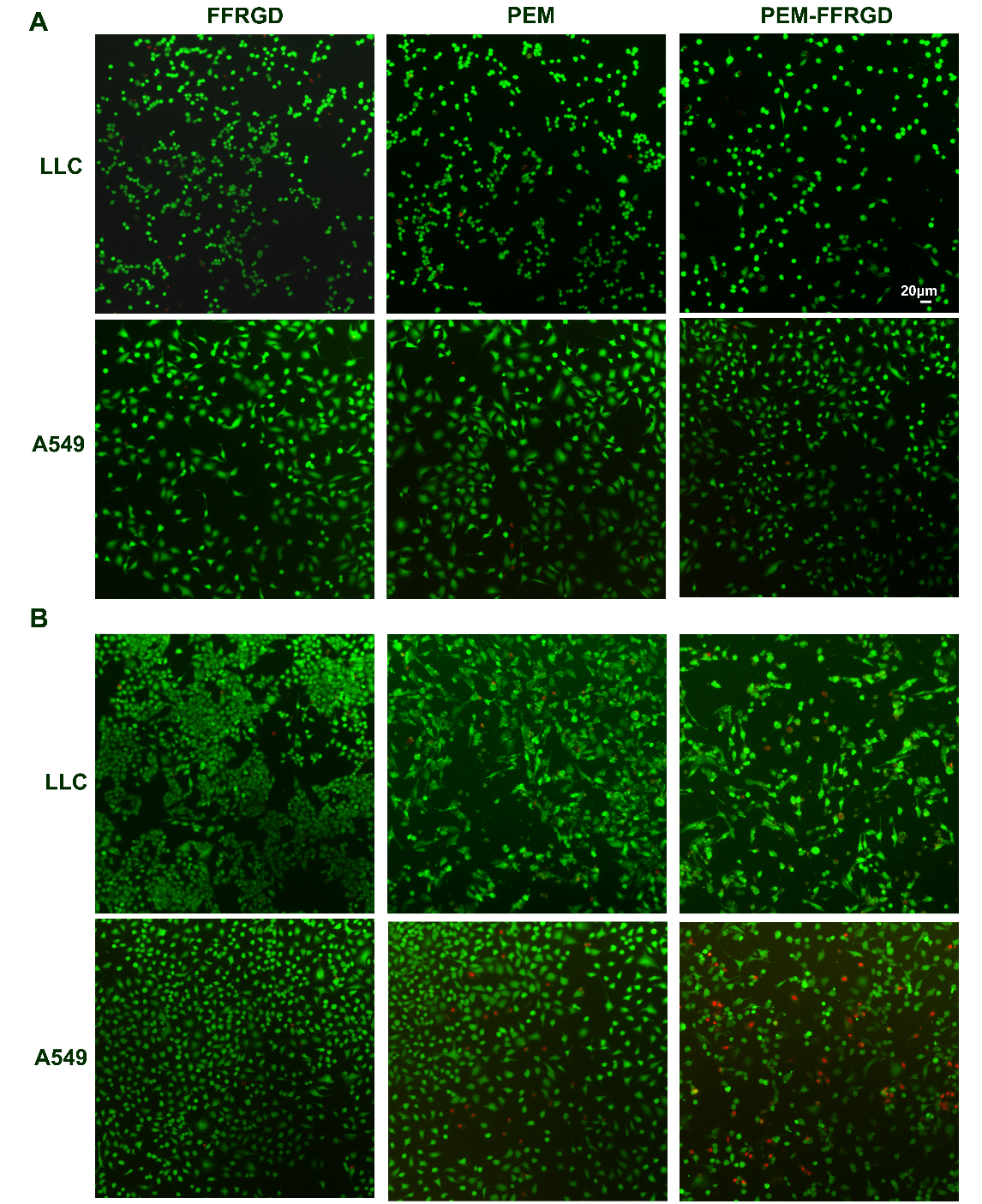


**FIGURE S4∣**The cytotoxicity of the PEM-FFRGD nanoparticle was determined using live/dead assays. **(A)** LLC cells and A549 cells were exposed to 6 μM and 12 μM FFRGD, PEM and PEM-FFRGD for 24 h and then imaged using microscopy. Scale bars, 20 µm. **(B)** LLC cells and A549 cells were exposed to 6 μM and 12 μM FFRGD, PEM and PEM-FFRGD for 48 h and then imaged using microscopy. Scale bars, 20 µm.
